# Comparison of the Clinical Features of SARS-CoV-2, Other Coronavirus and Influenza Infections in Infants Less Than 1-Year-Old

**DOI:** 10.1097/INF.0000000000002705

**Published:** 2020-04-27

**Authors:** Philippe Vanhems, Hubert Endtz, Cédric Dananché, Florence Komurian-Pradel, Valentina Sanchez Picot

**Affiliations:** 1 Hospices Civils de Lyon et Centre International de Recherche en Infectiologue, Lyon, France; 2 Emerging Pathogens Laboratory, Mérieux Foundation, Lyon, France and Medische Microbiologie en Infectieziekten (MMIZ), Erasmus MC, Rotterdam, The Netherlands; 3 Hospices Civils de Lyon et Centre International de Recherche en Infectiologue, Lyon, France; 4 Emerging Pathogens Laboratory, Mérieux Foundation, Lyon, France

## To the Editors:

We read with attention the review of Zimmerman and Curtis^1^ on Coronavirus Disease 2019 (COVID-19) among children and take the opportunity of this letter to share additional information. Infection with severe acute respiratory syndrome coronavirus 2 has mostly been reported in adults, though a recent publication described 9 infants <1-year-old with COVID-19.^2^ Among infant data are very few, though comparisons with infections due to other coronavirus strains will be helpful. The Pneumo-Study^3^ on the etiologic agents of pneumonia in children <5-year-old conducted by the Merieux Foundation Global Approach to Biological Research, Infectious diseases and Epidemics in Low-income countries (GABRIEL) network provides opportunities for comparisons.

We compared the published clinical features of hospitalized infants with COVID-19^2^ and hospitalized infants infected with other coronavirus strains or influenza from the GABRIEL project. The incident case-control Pneumo-study was done in children less than 5 in low-/middle-income countries between 2010 and 2014. The protocol and initial results are detailed elsewhere.^3,4^ The population was restricted to infants <1-year-old with features of pneumonia (ie, cases).^3^ Nasopharyngeal swabs were collected at admission to identify bacteria and viruses by reverse-transcription polymerase chain reaction (RT-PCR). Statistics were restricted to the same variables used by Wei et al^2^ and to cases with positive swabs for a coronavirus or influenza virus.

Of the 333 infants with pneumonia, 17 had CoV-positive nasopharyngeal swabs [7 (41.2%) with HKU1, 5 (29.4%) with CoV OC43, 3 (17.7%) with CoV NL63, 2 (11.8%) with CoV 229E] and 31 had an influenza-positive swab [22 (71%) with Influenza A, 9 (29%) with Influenza B]. Cough seems less prevalent in COVID-19 compared with other infected infants (Table 1). While no deaths occurred in infants with COVID-19,^2^ 3 infants infected with CoV in Pneumo-study died, 2 of whom were co-infected with *Streptococcus pneumoniae*.

**TABLE 1.**
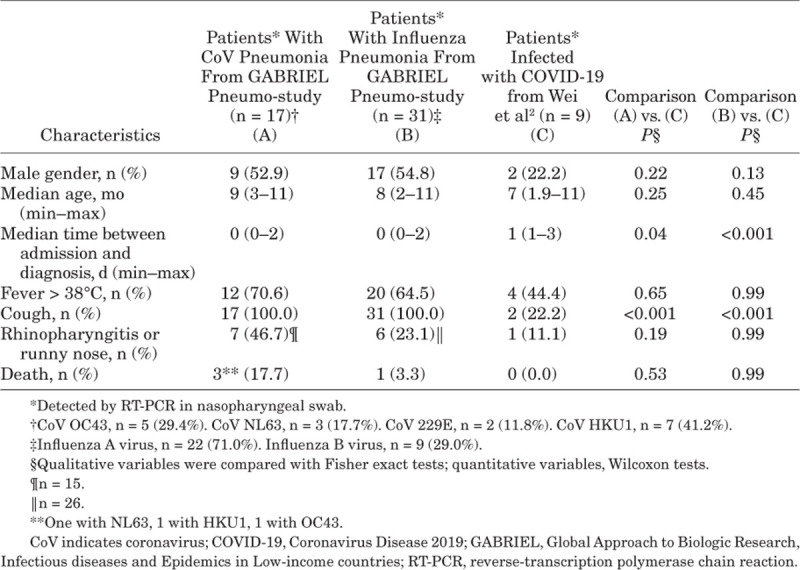
Comparison of the Characteristics of Coronavirus Disease 2019, Other Coronavirus and Influenza Infections Among Infants < 1-yr-old

This report underscores the lack of major differences in the clinical features of severe acute respiratory syndrome coronavirus 2 and other types of CoV or influenza infections among infants despite limited clinical features reported. COVID-19 infection does not seem more severe than other CoV or influenza infections in this population, possibly as all infect Angiotensin-Converting Enzyme 2 receptors in the upper airways. As influenza,^5^ the contribution of infants to the spread COVID-19 should be investigated. S. pneumoniae was co-detected in the CoV-infected infants who died in Pneumo-study while bacterial co-detection was not reported by Wei et al.^2^ Infants in both studies^2,3^ were hospitalized limiting selection bias but small sample sizes weakened statistical power. The incidence of COVID-19 in infants less than 1-year-old is currently low, but studies are needed to describe the clinical features, prognosis and impact of infected infants on the COVID-19 spread.

## ACKNOWLEDGMENTS

*Pneumonia Study GABRIEL members: Gláucia Paranhos-Baccalà, Shally Awasthi, Mélina Messaoudi Ashish Bavdekar, Jianwei Wang, Lili Ren, Sonali Sanghavi, Souleymane Diallo, Monidarin Chou, Tekchheng Eap, Mala Rakoto-Andrianarivelo, Muriel Maeder, Budragchaagiin Dash-Yandag, Wilma Basualdo, Pagbajabyn Nymadawa, Jean-William Pape, Vanessa Rouzier, Graciela Russomando, Mariam Sylla.

**Philippe Vanhems, MD, PhD** Hospices Civils de Lyon et Centre International de Recherche en Infectiologue Lyon, France**Hubert Endtz, MD** Emerging Pathogens Laboratory, Mérieux Foundation Lyon, France and Medische Microbiologie en Infectieziekten (MMIZ), Erasmus MC, Rotterdam, The Netherlands**Cédric Dananché, DrPharm, PhD** Hospices Civils de Lyon et Centre International de Recherche en Infectiologue Lyon, France**Florence Komurian-Pradel, PhD** **Valentina Sanchez Picot, DVM** Emerging Pathogens Laboratory Mérieux Foundation Lyon, France For the Pneumonia Study GABRIEL members*
